# Plant RNA Regulatory Network and RNA Granules in Virus Infection

**DOI:** 10.3389/fpls.2017.02093

**Published:** 2017-12-11

**Authors:** Kristiina Mäkinen, Andres Lõhmus, Maija Pollari

**Affiliations:** Department of Food and Environmental Sciences, Viikki Plant Science Centre, University of Helsinki, Helsinki, Finland

**Keywords:** RNA Interference, nonsense mediated mRNA decay, mRNA decay, processing bodies, stress granules, siRNA bodies, plant viruses

## Abstract

Regulation of post-transcriptional gene expression on mRNA level in eukaryotic cells includes translocation, translation, translational repression, storage, mRNA decay, RNA silencing, and nonsense-mediated decay. These processes are associated with various RNA-binding proteins and cytoplasmic ribonucleoprotein complexes many of which are conserved across eukaryotes. Microscopically visible aggregations formed by ribonucleoprotein complexes are termed RNA granules. Stress granules where the translationally inactive mRNAs are stored and processing bodies where mRNA decay may occur present the most studied RNA granule types. Diverse RNP-granules are increasingly being assigned important roles in viral infections. Although the majority of the molecular level studies on the role of RNA granules in viral translation and replication have been conducted in mammalian systems, some studies link also plant virus infection to RNA granules. An increasing body of evidence indicates that plant viruses require components of stress granules and processing bodies for their replication and translation, but how extensively the cellular mRNA regulatory network is utilized by plant viruses has remained largely enigmatic. Antiviral RNA silencing, which is an important regulator of viral RNA stability and expression in plants, is commonly counteracted by viral suppressors of RNA silencing. Some of the RNA silencing suppressors localize to cellular RNA granules and have been proposed to carry out their suppression functions there. Moreover, plant nucleotide-binding leucine-rich repeat protein-mediated virus resistance has been linked to enhanced processing body formation and translational repression of viral RNA. Many interesting questions relate to how the pathways of antiviral RNA silencing leading to viral RNA degradation and/or repression of translation, suppression of RNA silencing and viral RNA translation converge in plants and how different RNA granules and their individual components contribute to these processes. In this review we discuss the roles of cellular RNA regulatory mechanisms and RNA granules in plant virus infection in the light of current knowledge and compare the findings to those made in animal virus studies.

## Introduction

**Figure 1:**
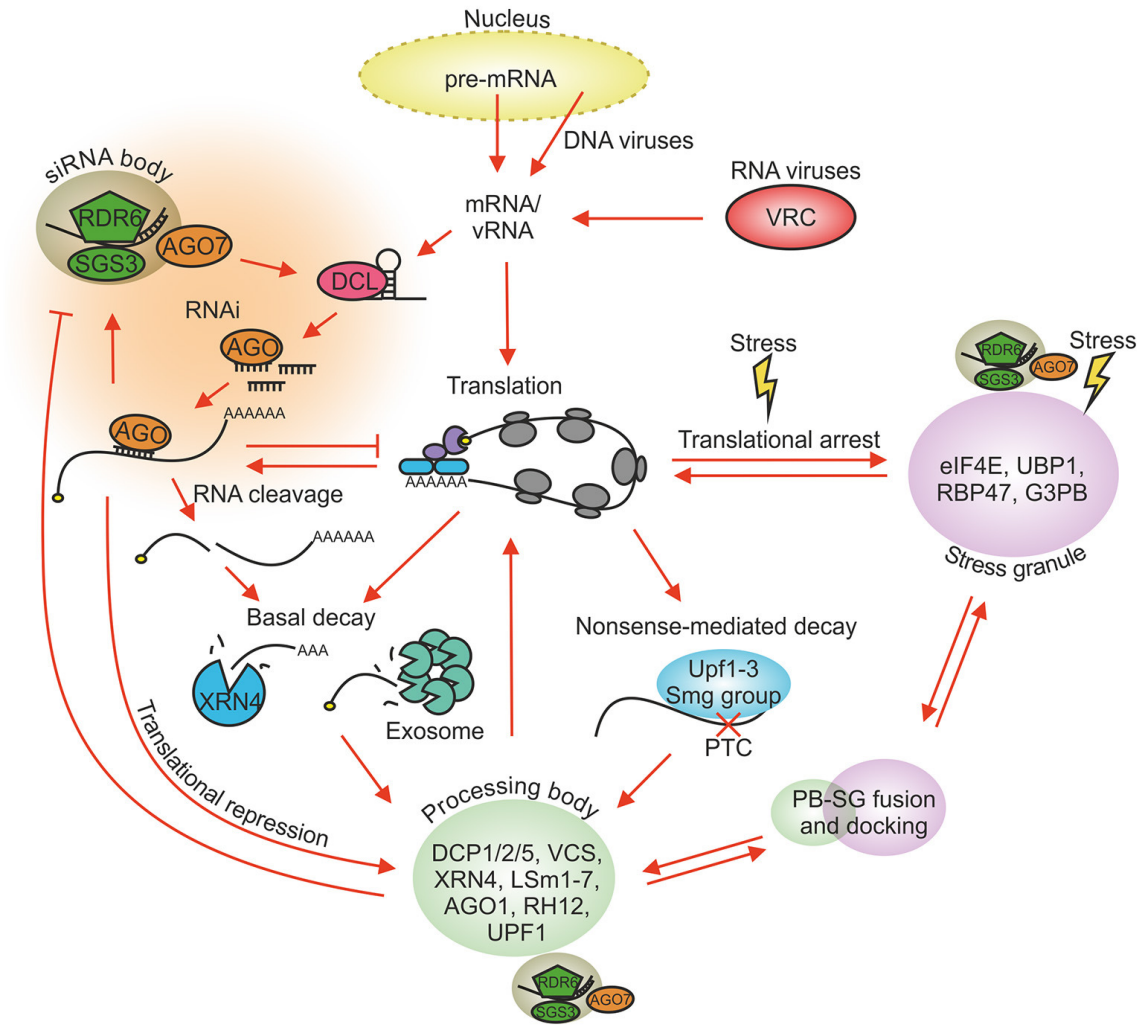
Major RNA regulatory processes in plant cells. Host mRNAs carrying the 5′ cap and the 3′ poly(A) tail are recruited to ribosomes for translation. vRNAs have evolved means to regulate host translation for their benefit. Various cellular stresses create SGs and increase the size and number of PBs. Translationally stalled pre-initiation complexes condense into SGs and can be released back to translation when stress conditions disappear. RNAs lacking the 5′ cap and the 3′ poly(A) tail are targeted for decay whereas translationally repressed RNAs can be redirected from PBs back to translation. PBs and SGs dock, fuse and exchange material. vRNAs and mRNAs recognized by the RNA silencing and NMD machineries are targeted for degradation. The RNA silencing functions are partially located to siRNA bodies and NMD to PBs. siRNA bodies can associate with PBs and SGs during stress. Only the main components of each granule type are shown. PB, processing body; SG, stress granule; NMD, nonsense-mediated decay; PTC, premature termination codon; VRC, virus replication complex.

Endogenous mRNAs produced in the nucleus of eukaryotic cells are subsequently exported to the cytoplasm for protein synthesis. There they are exposed to a complex RNA regulatory network. This network is able to adjust the amount of individual mRNAs within the total RNA population and to keep translationally active and inactive mRNAs in a delicate balance. Regulation of RNA allocation and turnover concerns not only mRNAs but also other RNA types in the cells including intruding viral RNAs (vRNAs). vRNAs possess features of aberrant RNAs which are often recognized as targets of the RNA decay machinery. Typically virus infection is initiated by the production of viral replication proteins and establishment of the replication complex in which progeny genomes and subgenomic RNAs (sgRNAs) are transcribed. Newly synthesized vRNAs and sgRNAs are translated into various viral proteins which carry out all viral functions together with compatible host factors. They also fight against the host's defense pathways. Therefore, to achieve a robust and productive infection, the vRNA molecules need to be able to cope with the cellular degradation and translation machineries. The eukaryotic RNA regulatory network including antiviral defense involves several complex pathways that have been shown to interact, compete and overlap on many levels (Figure 1) and altogether have a central role in maintaining cellular homeostasis. This review aims to illuminate the role of the RNA regulatory network in plant virus infection from different angles. We will overview the essential processes, cover the sites for these processes, the RNA granules, and highlight the ways some plant viruses modify and take advantage of the RNA regulatory network and its components.

## Plant RNA metabolic processes and RNA granules

### The general mRNA decay pathway

Functional endogenous mRNAs carrying the 5′ cap and the 3′ poly(A) tail are in general stable and translatable. They all have their individual turnover rates, some being long-lived and some taken to degradation within minutes. A constant exchange of protein factors within the messenger ribonucleoprotein (mRNP)-complexes assembling around the transcripts ensures the correct fate for each mRNA molecule during their dynamic life span (Mitchell and Parker, 2014). mRNA decay is typically initiated by shortening of the 3′ poly(A) tail by deadenylases and removal of the 5′ cap by decapping protein 2 (DCP2). The core mRNA decay machinery involves 5′–3′ decay by an XRN family exoribonuclease and 3′–5′ degradation by the exosome, which is a protein complex combining both exo- and endoribonuclease activities (reviewed in Garneau et al., 2007). The interaction and aggregation domains of the mRNA binding proteins enable the assembly of mRNPs into higher-order structures (Mitchell and Parker, 2014). When these assemblies become visible by light microscopy they are called RNA granules. Deadenylation, decapping, and degradation activities coalesce in RNA granules known as processing bodies (PBs; reviewed in Eulalio et al., 2007a). While PB components are indispensable for mRNA decay, aggregation into microscopically visible PBs is not a prerequisite for their function, also smaller entities can function in the diffuse cytoplasm (Eulalio et al., 2007b). *Arabidopsis* XRN exoribonucleases participate not only in mRNA decay but also in the degradation and processing pathways of rRNAs, miRNAs and other small RNAs (reviewed in Kurihara, 2017) and in vRNA degradation. Overexpression of XRN1 leads to enhanced degradation of tomato bushy stunt virus (genus *Tombusvirus*) RNA in yeast and cucumber necrosis virus (genus *Tombusvirus*) RNA in *Nicotiana benthamiana* (Cheng et al., 2007), whereas XRN4 knock-down facilitated systemic tobacco mosaic virus (genus *Tobamovirus*) infection in *N. benthamiana* (Peng et al., 2011). Based on these results XRN4 can be proposed to contribute to the antiviral defense *in planta*.

### Specialized decay pathways: nonsense-mediated decay

In eukaryotic organisms endogenous aberrant mRNAs containing premature in-frame stop codons are caught by the cellular quality control and degradation pathway known as nonsense-mediated decay (NMD; reviewed in He and Jacobson, 2015). The helicase Up frameshift 1 (UPF1) is a key component of the NMD machinery. In addition the complex comprises UPF2, UPF3 and at least one member of the Smg-group of proteins recruited to the activated complex to mediate target mRNA degradation. NMD complexes form co-translationally but also after the translocation of the targeted transcript to PBs for decay (Sheth and Parker, 2003). In addition to its surveillance function NMD functions in cellular stress responses. Many viruses express part of their open reading frames from sgRNAs and because of this expression strategy the genomic RNAs contain stop codons followed by atypically long 3′UTRs. Such vRNAs can be recognized and restricted by NMD not only in animals but also in plants. Accordingly, an increase in potato virus X (PVX; genus *Potexvirus*) and turnip crinkle virus (genus *Carmovirus*) infection was detected in *N. benthamiana* plants expressing transiently a dominant negative mutant UPF1 protein (Garcia et al., 2014). These two viruses belong to different superfamiles, PVX to Alphavirus-like and turnip crinkle virus to Flavivirus-like viruses. Studies with another (+)RNA virus from the Alphavirus-like superfamily, the human pathogen Semliki Forest virus (genus *Alphavirus*), indicated that depletion of NMD components UPF1, Smg5 and Smg7 increase the level of replication and viral protein production in human cells (Balistreri et al., 2014). Some (+)RNA virus genera, such as potyviruses, belonging to Picornavirus-like superfamily, lack sgRNAs and other NMD-eliciting signals from their genomes and therefore are most likely not recognized by this pathway (Garcia et al., 2014). NMD was proposed to affect vRNA early in the infection when RNA silencing is not yet induced and to be later inhibited by the infection with the aid of a viral protein or by some other *cis*- and *trans*-acting factors suppressing NMD (Garcia et al., 2014). In line with this suggestion, the C-terminal part of the Semliki Forest virus nsP3 protein was found to be required for protection of vRNA against UPF1-mediated degradation (Balistreri et al., 2014). Further investigations are required to elucidate the viral mechanisms for avoiding recognition and degradation of vRNA by NMD.

### Specialized decay pathways: RNA silencing pathway

The third pathway actively in use *in planta* to regulate gene expression and RNA stability is RNA silencing (reviewed recently in Csorba et al., 2015). This evolutionarily conserved mechanism in eukaryotes is triggered by double-stranded RNAs (dsRNA). Although RNA silencing contributes to many cellular functions, antiviral defense has been proposed to be its indigenous function (Pumplin and Voinnet, 2013). RNA silencing involving endonucleolytic cleavage and/or translational repression of the target RNAs is regulated by small RNAs (sRNAs) consisting of many types of 20–24 nucleotide-long dsRNA molecules. sRNAs produced during viral infection, viral small interfering RNAs (vsiRNAs), restrict the corresponding virus in a sequence-specific manner. VsiRNA production is catalyzed by DICER-LIKE (DCL) endonucleases and dsRNA-binding (DRB) proteins. During the effector step of RNA silencing vsiRNAs form RNA-induced silencing complexes (RISCs) with ARGONAUTE (AGO) proteins to cleave or translationally repress the target RNAs guided by the complementarity of the vsiRNA (Figure 1). Plant-specific RNA-dependent RNA polymerases (RDRs) together with cofactors SUPPRESSOR OF GENE SILENCING 3 (SGS3) and SILENCING DEFECTIVE 5 convert single-stranded RNA to double-stranded RNA (dsRNA) for their subsequent cleavage to siRNAs. RDR6 and SGS3 contribute also to the amplification step of RNA silencing by catalyzing dsRNA synthesis from the RISC cleavage products. RDR6, SGS3, and AGO7, which function in trans-acting siRNA (tasiRNA) synthesis, all have roles in plant defense against virus infections (Mourrain et al., 2000; Qu et al., 2008) and they aggregate in cytoplasmic bodies called siRNA bodies (Jouannet et al., 2012; Moreno et al., 2013). As RNA silencing is the major plant antiviral mechanism all plant viruses need to have mechanisms to overcome it. Viral suppressors of RNA silencing VSRs have been identified from both plant DNA and RNA viruses and they provide protection for vRNA by multiple mechanisms including sequestration of vsiRNAs to prevent RISC assembly and by interfering with DCL and RISC activities, AGO loading, systemic silencing and amplification of silencing (Csorba et al., 2015).

In contrast to the vast knowledge about RISC-mediated slicing of vRNA, much less is known about sRNA-guided repression of vRNA translation in plants. RISC-mediated translational repression by endogenous miRNAs is common in plants (Brodersen et al., 2008) and, similarly to the situation in animal cells, requires the association of sRNAs, their mRNA targets and AGO proteins with ribosomes (Lanet et al., 2009; Reynoso et al., 2013; Fatyol et al., 2016). sRNA-mediated translational repression may also restrict vRNA translation *in planta*. sRNA with a perfect complementarity to a target sequence in the 5′ untranslated region (UTR) of the tobacco etch virus (genus *Potyvirus*) inhibited translation (Iwakawa and Tomari, 2013). In this study the tobacco etch virus 5′UTR was part of a monocistronic reporter gene mRNA leaving the role of RISC-mediated translation repression in plant virus infection for future studies. AGO1 association with ribosomes is held as a hallmark of translational repression (Lanet et al., 2009). As HCPro, the RNA silencing suppressor of potyviruses, and AGO1 interact and associate with ribosomes *in planta* it was proposed that HCPro may counteract translational repression during potyvirus infection (Ivanov et al., 2016). AGO1 is involved in translational repression of RNA2 associated with recovery of *Nicotiana benthamiana* from tomato ringspot virus infection (Ghoshal and Sanfaçon, 2014; Karran and Sanfacon, 2014). Recovery from tobacco rattle virus infection which normally occurs in *Arabidopsis* does not take place in the presence of a strong VSR, P38, encoded by turnip crinkle virus, which also suggests involvement of RNA silencing in the translational repression of viral RNA in the recovered plants (Ma et al., 2015).

### RNA storage during stress

The cellular RNA regulatory network is able to react to various conditions such as oxidative and heat stress and nutrient depletion by reducing overall translational rate and storing selected mRNAs for further use after the cell has been released from stress. Translational shutdown is thought to be an ancient mechanism that enables cells to selectively redirect resources to survival in adverse conditions. In animal cells stress-induced phosphorylation of the eukaryotic initiation factor eIF2α is a central trigger for the formation of subcellular structures called stress granules (SGs). SGs are sites where the already assembled ribosomal 43S and 48S pre-initiation complexes are relocated for storage (reviewed in Lloyd, 2013). Among the kinases phosphorylating eIF2α during cellular stress in animal cells are general control nondepressible 2 kinase (GCN2), double-stranded RNA (dsRNA)-activated protein kinase R (PKR) and PKR-like endoplastic reticulum kinase (PERK). eIF2α phosphorylation interferes with eIF2α-GDP recycling leading to compromised availability of eIF2α-GTP-Met-tRNAiMet and an overall reduction of translation (Kedersha et al., 1999, 2002; Kimball et al., 2003). The ensuing translational shutdown rapidly triggers the aggregation of halted pre-initiation complexes into SGs for sorting and temporary storage. *In planta* eIF2α phosphorylation is induced e.g., upon UV-induced stress (Meteignier et al., 2016) and wounding (Lageix et al., 2008), by activation of GCN2 kinase in the latter case. Activation of PKR and subsequent eIF2α phosphorylation is a common antiviral response leading to global translational repression during many animal virus infections (see examples in Lloyd, 2013), but there is no direct evidence for PKR-mediated antiviral defense in plants. A mammalian protein P58, which contains several tetratricopeptide repeat motifs and a conserved J domain of the DnaJ chaperone, acts as an inhibitor of PKR. It is recruited by the influenza virus to limit PKR-mediated antiviral responses (Melville et al., 2000). Similarly to the mammalian P58 protein its plant homolog P58^IPK^ was proposed to regulate PKR- or PERK-like kinases during virus infection (Bilgin et al., 2003). An increase in eIF2α phosphorylation was detected in P58^IPK^-silenced *N. benthamiana* plants upon virus infection (Bilgin et al., 2003), but apart from this study the evidence for a PKR-mediated antiviral response is non-existing. No sequence homologs of the animal PKR can be identified from the *N. benthamiana* genome. No eIF2α phosphorylation could be detected either in connection to PVX infection or during resistance gene-mediated antiviral defense response against PVX associated with repression of viral translation in *N. benthamiana* (Meteignier et al., 2016). Furthermore, eIF2α phosphorylation was not detected either in turnip crinkle virus or turnip yellow mosaic virus-infected *Arabidopsis* (Zhang Y. et al., 2008). Although evidence for involvement of SG components in plant virus infection exist (Hafrén et al., 2015; Krapp et al., 2017), the molecular basis of SG induction during virus infection is an uncovered area *in planta*.

### Crosstalk between different RNA processes and RNA granules

Many RNA regulatory processes share common protein factors and often co-localize to the same subcellular sites. In addition to the mRNA decay-associated proteins PBs contain proteins involved in mRNA surveillance (NMD-associated proteins), RNA silencing-associated proteins and proteins regulating translational repression (Eulalio et al., 2007a). A spatial overlap of the RNA silencing proteins AGO1 and SILENCING DEFECTIVE 3 within *Arabidopsis* PBs was demonstrated (Xu and Chua, 2011). In animal cells PBs assemble upon induction of gene silencing and knock-down of the AGO proteins prevents PB formation (Eulalio et al., 2007b). The mechanisms of mRNA decay and RNA silencing are functionally coupled also *in planta* (Gazzani et al., 2004). The relationship is antagonistic since these pathways compete for the same RNA substrates lacking the 5′ cap or the 3′ poly-A signal (Moreno et al., 2013; Martinez de Alba et al., 2015). These RNA molecules can either undergo exonucleolytic RNA decay or re-enter the RNA silencing pathway through RDR6-catalyzed dsRNA synthesis. It has been proposed that RNA decay can prevent the initiation of threshold-dependent RNA silencing by degrading ssRNA substrates before aberrant RNAs accumulate to critical levels (Garcia et al., 2014). Indeed, many proteins of the RNA quality control machinery, involved both in 5′–3′ and 3′–5′ RNA degradation, have been identified as endogenous RNA silencing suppressors. For example, in *Arabidopsis* the PB components DCP1, DCP2, and VARICOSE (VCS) have been shown to suppress RDR6-dependent post-transcriptional gene silencing (PTGS) (Thran et al., 2012; Martinez de Alba et al., 2015). Endogenous silencing suppression activity has also been shown for *Arabidopsis* FIERY1, XRN2, and XRN3 (Gy et al., 2007) and the exosome core subunits RRP4 and RRP41 or exosome co-factors RRP44A, RRP6L1, and HEN2 (Moreno et al., 2013; Lange et al., 2014; Hematy et al., 2016). Mutations in XRN4 (Gazzani et al., 2004) or DCP2 (Thran et al., 2012) enhance PTGS. Furthermore, it has been shown that a dysfunctional decapping pathway leads to RDR6-dependent production of siRNAs against endogenous protein encoding mRNAs, causing considerable changes in the transcriptome. And in turn, *Arabidopsis* PTGS mutants were able to rescue the drastic transcriptome alterations caused by the dysfunctional mRNA decay pathway (Martinez de Alba et al., 2015). Garcia et al. (2014) also suggested for exogenous RNAs that saturation of NMD by vRNAs may switch the regulation toward RDR action and siRNA-mediated silencing of viral transcripts.

**Figure 2:**
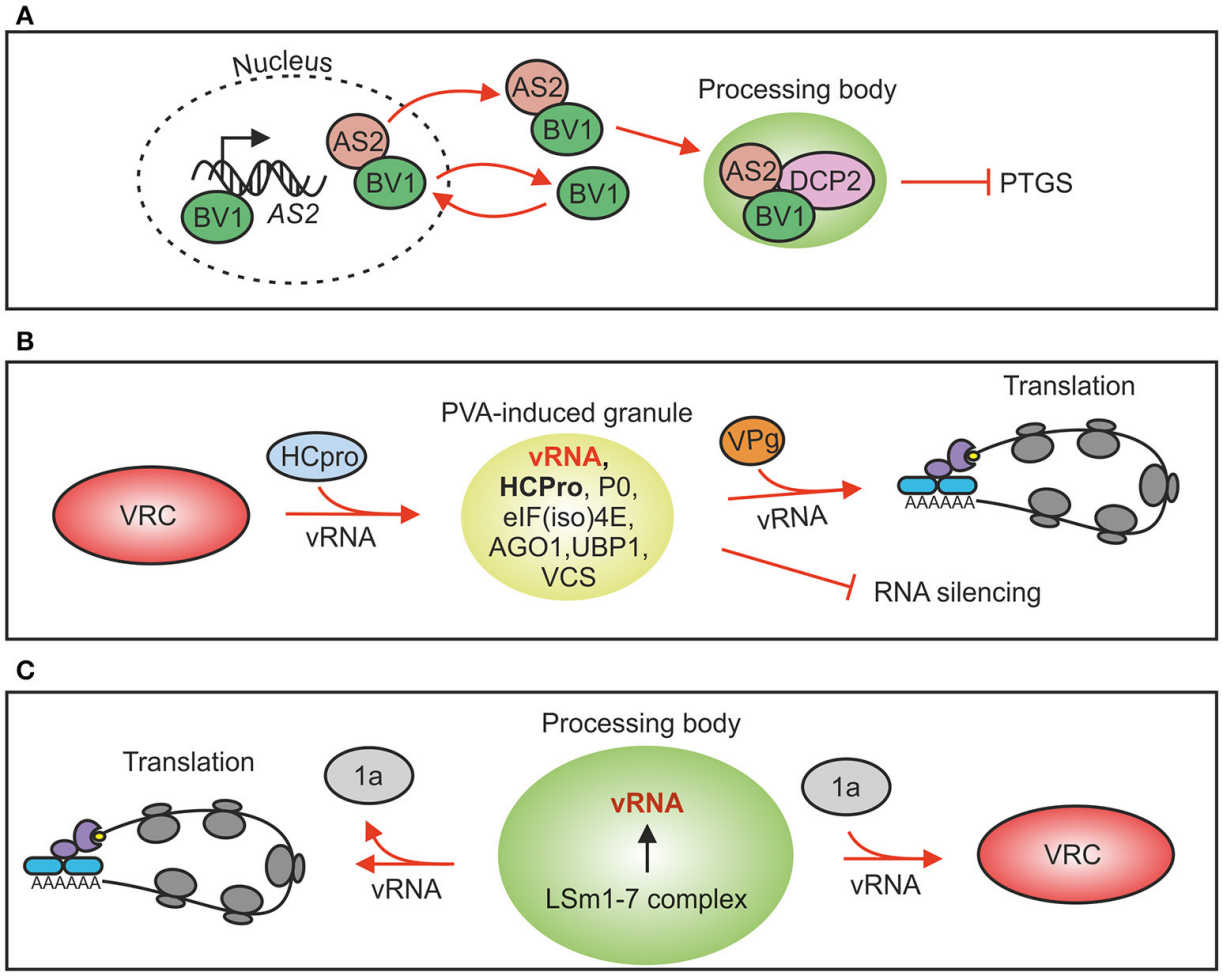
Plant virus interactions with RNP granules. **(A)** Cabbage leaf curl virus nuclear-cytosol shuttle protein BV1 can bind to the promoter region of *AS2* gene and induce its expression. AS2 is transported with BV1 to PBs where it activates DCP2-mediated decapping. Increased RNA decay down-regulates RNA silencing and consequently virus infection gets advantage. **(B)** Potato virus A silencing suppressor protein HCPro induces formation of RNP granules that contain viral RNA, ribosomal protein P0 and several PB and SG markers. Viral protein genome-linked, VPg, abolishes PVA-induced granules and increases viral translation. Evidence to support silencing suppression-related PG functions exists. **(C)** Brome mosaic virus genomic RNAs 2 and 3 contain motifs for binding to the decapping activator LSm1-7 complex in PBs of yeast. This interaction is required both for brome mosaic virus translation and replication. In the absence of viral protein 1a vRNA is subjected to translation whereas in its presence vRNA is recruited to replication.

Plant protein ASYMMETRIC LEAVES 2 (AS2) is a PB component and an activator of DCP2-mediated decapping. It also inhibits siRNA production and functions as an endogenous PTGS suppressor (Ye et al., 2015). AS2 expression is upregulated by the cabbage leaf curl virus (genus *Begomovirus*) nuclear shuttle protein BV1 which also causes AS2 to exit the nucleus. In the cytoplasm AS2 activates decapping by DCP2, reduces siRNA production, weakens RNAi and promotes virus infection (Ye et al., 2015; Figure 2A). The authors proposed that this strategy could be used also by other viruses to weaken antiviral defense both in plant and animal hosts. A recent work (Conti et al., 2017) supports this proposal by providing evidence that both tobacco mosaic virus coat protein and movement protein may have evolved to interfere with RNA decay by increasing it in order to down-regulate RNA silencing against its genomic RNA.

In sense RNA-mediated silencing *in planta* RdRs copy ssRNA to dsRNA, which are subsequently process into siRNAs by DCLs. This process takes place in cytoplasmic siRNA bodies (Fukunaga and Doudna, 2009; Kumakura et al., 2009). Interestingly, upon heat stress the number of siRNA bodies increases remarkably and these structures become positive for SG markers in addition to the SGS3 and AGO7 markers (Jouannet et al., 2012). This suggests that mRNAs stalled in translation may accumulate in cytoplasmic foci representing fusions between SGs and siRNA bodies. The tobacco etch virus 6K2 protein is an inducer of viral replication complex formation (Schaad et al., 1997). A partial (60%) spatial overlap of the tobacco etch virus 6K2 and AGO7 was detected proposing a link between potyviral replication-associated membranous structures and siRNA bodies (Jouannet et al., 2012). This finding could represent a point of convergence between viral replication and the host's defense mechanisms.

Rather than being fixed entities SGs have emerged as dynamic structures that actively exchange both proteins and mRNPs with the cellular environment (Mollet et al., 2008; Weber et al., 2008; Sorenson and Bailey-Serres, 2014). SGs could be the first location for mRNAs after translation has halted since mRNAs in SGs are still associated with translation initiation factors, but based on studies in yeast it is also possible that mRNPs from PBs may enter SGs (Buchan and Parker, 2009). Translationally inactive mRNAs together with their associated proteins and regulatory RNAs accumulates into PBs. The eukaryotic mRNA cycle model about sorting mRNAs between translation, storage and degradation in SGs and PBs, termed the mRNA cycle, was set forth by Balagopal and Parker (2009). PBs and SGs have been shown to fuse in animal cells (Kedersha et al., 2005; Thomas et al., 2011; Buchan et al., 2013), which suggests that markers commonly distinguishing PBs from SGs may overlap in RNA granules formed under certain stress conditions. For example potato virus A (PVA; genus *Potyvirus*) -induced RNA granules contain both OLIGOURIDYLATE BINDING-PROTEIN 1 (UBP1) of plant SGs and VCS and AGO1 of PBs (Hafrén et al., 2015) showing that these granules cannot be classed unambiguously into PBs or SGs but that they have a unique composition defined by PVA infection (Figure 2B). The main distinction between SGs and PBs is that SGs contain translation factors and PBs mRNA decay factors. These examples highlight the wide crosstalk between regulatory pathways and active exchange of protein components between the associated subcellular structures.

## Plant RNA granule proteins

More than 70 different proteins have been identified in the SGs and over 20 in the PBs of eukaryotic cells. In addition many proteins are shared between different RNA granule types in yeast and mammals (Poblete-Durán et al., 2016). Although it is evident that plant, yeast and animal PBs and SGs have similar functions (Xu et al., 2006; Weber et al., 2008; Xu and Chua, 2009; Sorenson and Bailey-Serres, 2014), the composition of RNA granules is currently less studied in plants. Nevertheless, several hallmark-proteins have been identified from plant SGs, including UBP1, eukaryotic initiation factor 4E (eIF4E), poly-A binding protein (PABP), and PBs, including DCP1, DCP2, VCS, and AGO1 (Xu et al., 2006; Weber et al., 2008; Sorenson and Bailey-Serres, 2014).

### Plant SG proteins

T-cell intracellular antigen 1 (TIA-1), Ras-GAP SH3 domain-binding protein (G3BP), and tristetraprolin (TTP) are among the RNA-binding proteins promoting SG formation in animal cells (reviewed in Kedersha et al., 2013). In plants UBP1, RNA-binding proteins 45 and 47 (RBP45/47), and PABP represent proteins related to animal TIA-1 (Sorenson and Bailey-Serres, 2014). TIA-1's capacity to promote SG formation in animal cells depends on the phosphorylation of eIF2α as well as its high-affinity RNA-binding domain and a glutamine-rich prion-like domain with self-aggregation properties (Kedersha et al., 1999; Gilks et al., 2004). Likewise plant UBP1 contains several RNA-binding domains and a prion domain which are essential for SG aggregation. In *Arabidopsis* the removal of these domains from UBP1 or RBP47 crippled the assembly of SGs (Weber et al., 2008) and as can be expected from a SG protein, plant UBP1 is a protein mainly associated with translationally silent mRNAs (Sorenson and Bailey-Serres, 2014). Recent studies in plant systems have highlighted UBP1's functional role in stress-related post-transcriptional gene regulation. For example in low oxygen conditions UBP1 preferentially bind translationally repressed mRNAs selectively aggregating them into SGs. Transcript residence time in SG's is brief and once oxygen is resupplied halted pre-initiation complexes are rapidly recycled into actively translating polysomes (Sorenson and Bailey-Serres, 2014).

Adding to a foundation built on eIF4E, UBP1, RBP47, TTP, and G3BP, several plant proteins have been identified as potential SG components based mainly on their stress-induced co-localization into cytoplasmic foci with one or more of the traditional SG markers (Table 1). Recent examples include members of the *Arabidopsis* tandem CCCH zinc finger protein family (Pomeranz et al., 2010; Bogamuwa and Jang, 2013, 2016; Qu et al., 2014), a stress-responsive C-terminal binding protein ANGUSTIFOLIA (Bhasin and Hulskamp, 2017), the CML38 calcium sensor (Lokdarshi et al., 2016) and an RNA-binding TUDOR-SN homolog which is important for the stabilization of transcripts encoding stress-related proteins (Yan et al., 2014; Krapp et al., 2017).

**Table 1:** Plant stress granule proteins and their animal counterparts.

**Protein component in plants**	**Animal homolog**	**Function**	**References**
EUKARYOTIC INITIATION FACTOR 4E (eIF4E)	eIF4E	Translation initiation	Kedersha et al., 2002; Weber et al., 2008
POLYADENYLATE-BINDING PROTEIN 47 (RBP47)	T-cell intracellular antigen TIA-1	SG assembly	Lorkovic et al., 2000; Weber et al., 2008
OLIGOURIDYLATE-BINDING PROTEIN 1 (UBP1)	T-cell intracellular antigen TIA-1	SG assembly	Weber et al., 2008; Sorenson and Bailey-Serres, 2014
RAS-GAP SH3 DOMAIN-BINDING PROTEIN (G3BP)	Ras-GAP SH3-domain–binding protein G3BP1	SG assembly	Krapp et al., 2017
POLY(A)-BINDING PROTEIN (PABP)	T-cell intracellular antigen TIA-1	Recruitment of poly-A mRNAs to SGs	Weber et al., 2008
CALMODULIN-LIKE 38 (CML38)	Calmodulin-like family	Calcium sensor, stress signaling	Lokdarshi et al., 2016
ANGUSTIFOLIA (AN)	C-terminal-binding protein/brefeldin A-ADP ribosylated substrate CtBP/BARS	Regulation of SG formation	Folkers et al., 2002; Bhasin and Hulskamp, 2017
TUDOR-SN (TSN)	TUDOR-SN	RNA binding and stabillization	Frei dit Frey et al., 2010; Yan et al., 2014
AtTZF1	Tristetraprolin TTP	Tandem zinc-finger protein, RNA delivery and protein recruitment into PBs and SGs	Pomeranz et al., 2010; Qu et al., 2014; Bogamuwa and Jang, 2016

### Plant PB proteins

In *Arabidopsis* the decapping complex comprises DCP1, DCP2 (TDT), DCP5, VCS, and possibly DEA(D/H)-box RNA helicase DHH1/AtRH12 (Xu et al., 2006; Xu and Chua, 2009). Human protein Ge-1/Hedls, an enhancer of mRNA decapping and a component of PBs, is thought to provide a scaffold for the decapping complex comprising DCP2 and the decapping co-activators DCP1, DEAD-box protein RCK/p54 and enhancer of decapping 3 (Fenger-Gron et al., 2005). VCS is the plant homolog of Ge-1/Hedls and serves as potential hub for protein-protein interactions in plants as well. VCS contains repeating WD-40 domains which typically coordinate the assembly of multiprotein complexes where they serve as scaffolds for protein-protein interactions. In PBs VCS's main role is to facilitate the interaction between the catalytic subunit DCP2 and its coactivator DCP1 (Xu et al., 2006). Similarly to animals VCS is also required for miRNA-guided translational repression in *Arabidopsis* (Brodersen et al., 2008). The plant-specific SUO is another PB-localized protein involved in translational repression (Yang et al., 2012). The SUO protein contains GW-repeats which potentially mediate interactions with AGO proteins that are central for miRNA-mediated translational repression. Mutations in SUO GW-repeats increased the abundance of several endogenous proteins without affecting their mRNA levels indicating release from translational repression (Yang et al., 2012). The functions of the PB-associated proteins thus suggest a role for PBs both in mRNA decapping and translational repression also in plants.

VCS/Ge1 appears to be an integral host factor for achieving virus infection. PVA infection redirects host VCS into RNA granules together with vRNA. The depletion of VCS disabled viral gene expression and reduced the amount of vRNA in infected cells suggesting that PVA uses VCS to its advantage (Hafrén et al., 2015). A similar effect was reported from human liver cells infected with Hepatitis C Virus: Ge-1 was required for optimal accumulation of vRNA and proteins (Pager et al., 2013).

In addition to the core components efficient decapping requires accessory proteins that associate with PBs to enhance or regulate mRNA turnover. In animals and plants alike the evolutionarily conserved Sm-like proteins (LSm1-7) form a heptameric structure that requires a protein-associated with topoisomerases (PAT1) to form a bridge between deadenylated mRNA and the decapping complex (Tharun, 2009; Golisz et al., 2013; Roux et al., 2015; Vindry et al., 2017). An *Arabidopsis lsm5* mutant was sensitive to drought, heat and salt stress (Golisz et al., 2013; Okamoto et al., 2016) while a *pat1* mutant displayed a constitutively activated innate immune response via an R-protein (Roux et al., 2015) emphasizing their considerable importance in optimal stress responses. The role of the tandem zinc finger protein TTP is not limited to SGs as it also promotes the assembly of PBs and activates decapping (Bogamuwa and Jang, 2016). Protein constituents of PBs in plant cells and their animal homologs are listed in Table 2.

**Table 2:** Plant processing body proteins and their animal counterparts.

**Protein component in plants**	**Animal homolog**	**Function**	**References**
DECAPPING PROTEIN 1 (DCP1)	DCP1	Coactivator of DCP2	Xu et al., 2006
DECAPPING PROTEIN 2 (DCP2)	DCP2	Catalytic subunit of the decapping complex	Xu et al., 2006
DECAPPING PROTEIN 5 (DCP5)	RAP55/LSm14A	Involved in PB assembly, decapping, and translational repression. Has a role in translational repression in Xenopus oocytes	Tanaka et al., 2006; Xu and Chua, 2009
VARICOSE(VCS)	EDC4/Ge-1/HEDSL	Scaffolding protein in the decapping complex. Enhancer of decapping, involved in translational repression	Xu et al., 2006; Goeres et al., 2007
EXORIBONUCLEASE 4 (XRN4)	XRN1	5′–3′ exonuclease	Rymarquis et al., 2011
AtRH12	DEA(D/H)-box RNA helicase DHH1p/CGH-1	Translational repression, PB dynamics, promotes mRNA decay (for retroviruses required for replication and encapsidation)	Xu et al., 2006; Carroll et al., 2011; Yu et al., 2011; Sweet et al., 2012
LIKE Sm 1-7 (LSm1-7)	LSm1-7	Enhancers of decapping, promotion of PB assembly	Golisz et al., 2013
PROTEIN ASSOCIATED WITH TOPOISOMERASES 1 (PAT1)	PatL1	Activates decapping and inhibits translation	Scheller et al., 2007
ARGONAUTE 1 (AGO1)	AGO1	miRNA-dependent endonuclease	Pomeranz et al., 2010
SUO	no known homologs	Interacts with AGO proteins in PBs to promote translational repression by miRNAs (Possible functional analog of GW182)	Yang et al., 2012
UP-FRAMESHIFT 1 (UPF1)	UPF1	Involved in nonsense-mediated mRNA decay processes	Merai et al., 2013
ASYMMETRIC LEAVES 2 (AS2)	no known homologs	Decapping activator, endogenous silencing suppressor	Ye et al., 2015
AtTZF1	Tristetraprolin TTP	Tandem zinc-finger protein, RNA delivery and protein recruitment into PBs and SGs	Pomeranz et al., 2010; Qu et al., 2014; Bogamuwa and Jang, 2016
SILENCING DEFECTIVE 3 (SDE3)	RISC Complex RNA Helicase MOV10	RNA helicase, possible functional analog of GW182	Dalmay et al., 2001; Xu and Chua, 2011

## SGs and plant virus infection

Multifunctional G3BPs participate in SG assembly upstream of eIF2α phosphorylation. In animal cells stress-induced dephosphorylation of S149 in G3BP causes a conformational change enabling homo- and heteromultimerization with G3BP2 (Tourriere et al., 2003; Matsuki et al., 2013). Oligomerization of G3BPs causes the nucleation of SGs even in the absence of stress suggesting it might be a prerequisite for the eventual assembly of other SG components (Matsuki et al., 2013). SGs are regarded as antiviral compartments and many viruses have developed means to disrupt SG assembly via G3BP interactions. Some viruses, such as vaccinia virus and hepatitis C virus, have co-opted G3BP to serve as a potential component of viral factories and replication complexes (Yi et al., 2006; Katsafanas and Moss, 2007) or an assisting factor for virion assembly (Garaigorta et al., 2012). Polioviral 3C protease cleaves G3BP leading to the formation of SGs without G3BP (White et al., 2007; Piotrowska et al., 2010). A G3BP-like protein localizing to plant SGs was recently discovered in *Arabidopsis*. AtG3BP was identified through its interaction with a viral protein, the nuclear shuttle protein 3 (NSP3) of the abutilon mosaic virus (genus *Begomovirus*), in SGs (Krapp et al., 2017). The interaction between G3BP and viral proteins was mapped to a conserved FGDF-type motif. Also in animal cells G3BP has been shown to associate with the C-terminal FGDF motifs of ns3 protein of the alphaviruses Semliki Forest virus and Chikungunya virus (Panas et al., 2014; Scholte et al., 2015). The FGDF motif is relevant for G3BP-interactions of the host proteins as well (Panas et al., 2015). Interestingly, FGDF motifs are present in the proteases of potyviruses, waikaviruses, and closteroviruses suggesting that SG regulation via G3BP interactions may have greater influence in plant virus infections than currently known.

Animal RNA viruses make use of TIA1, the homolog of UBP1. A recent report showed that the knockdown of TIA-1 impaired the accumulation of Newcastle disease virus (family *Paramyxovirdae*) in HeLa cells (Sun et al., 2017). Other viruses such as the Dengue virus (genus *Flavivirus*) and Poliovirus (PV; genus *Enterovirus*) have evolved a different strategy: viral proteins sequester TIA1 so that it cannot form functional SGs. The Dengue virus interferes with SG formation by using nonstructural protein NS1 to sequester TIA1 to the perinuclear space (Xia et al., 2015). Likewise the poliovirus disrupts host defense by aggregating TIA1 into nonfunctional foci (White and Lloyd, 2011; Fitzgerald and Semler, 2013).

*Nicotiana benthamiana* UBP1 was identified as a component of PVA-induced RNA granules (Hafrén et al., 2015). Although these granules do not represent canonical SGs, UBP1 is important for the formation of PVA-induced granules and seems to be one of the many host factors PVA employs to ensure a successful infection. Potyviral genome-linked protein VPg specifically promotes PVA translation (Eskelin et al., 2011; Hafrén et al., 2013). Interestingly, the granulation-prone UBP1 does not promote PVA translation together with VPg, whereas many other components co-localizing to PVA-induced granules, e.g., VCS and eIF(iso)4E, do. The role of these granule proteins in PVA translation in turn propose a link between the granules and viral translation. Supporting this, VPg is able to disrupt the PVA-induced granules while promoting translation (Hafrén et al., 2015). Additional evidence suggested a functional link between granule formation and RNA silencing suppression. PVA-induced granules were neither formed in the absence of potyviral silencing suppressor HCPro nor by HCPro silencing suppression-deficient mutants suggesting that PVA-induced granules could be the sites for HCPro-guided silencing-suppression function (Hafrén et al., 2015). Similar granule-like foci, observed in the vicinity of the ER and the microtubule cytoskeleton, were induced by the HCPro of potato virus Y in *Nicotiana benthamiana* (del Toro et al., 2014).

Cysteine3Histidine (CCCH)-type zinc finger proteins and tandem zinc finger proteins (TZF) comprise a large protein family well conserved across eukaryotes. The PB and SG component Tristetraprolin (TTP) belongs to this protein family. It has a critical role in mRNA metabolism in recruiting enzymes involved in deadenylation, decapping, and exonucleolytic activities to specific mRNAs related to innate immunity and inflammatory responses in animals proposing a role in virus infection (Hamid and Makeyev, 2016). In plants the CCCH-motif is preceded by an arginine-rich (RR)-motif and therefore these proteins are called RR-TZF proteins (reviewed in Bogamuwa and Jang, 2014). Although plant RR-TZF are involved in biotic stress responses potentially by binding to specific elements of the corresponding mRNAs and recruiting the assembled mRNP complexes into PBs and SGs, no report has linked them to regulation of virus infection in plant cells so far.

## PBs and plant virus infection

Although PBs have housekeeping functions and are always present in the cells, formation of visible PBs is activated for example as a consequence of RNA silencing leading to an increase in translationally repressed mRNAs in the cell (Eulalio et al., 2007b). PB assembly upon induction of gene silencing was shown in plants using an RNA hairpin targeting one specific transcript, the *Sulphur* mRNA (Meteignier et al., 2016) and in connection to virus recovery (Ma et al., 2015). Viruses can affect PBs either by dispersing PB structures and components or by interfering with the mechanism of PB formation. For example poliovirus and human Coxsackie virus, both belonging to family *Picornaviridae*, interfere with PB formation during their replication cycle (reviewed in Malinowska et al., 2016). In addition, polioviral 3C protease processes XRN1 and DCP1 (Dougherty et al., 2011). Some viruses exploit PB components for their benefit. The LSm1-7 complex, which is an activator of decapping in the 5′–3′ exoribonucleolytic pathway (Tharun et al., 2000) and a component of PBs (Ingelfinger et al., 2002), is a central regulator of brome mosaic virus (genus *Bromovirus*) translation and replication (Galao et al., 2010). Studies in yeast revealed that the *cis*-acting RNA replication signals within RNA 2 and RNA3 were found to direct these brome mosaic virus RNAs into PBs (Beckham et al., 2007). PBs create an environment which lacks the components of the translation machinery and subsequently may facilitate replication complex assembly. On this basis, a role for PBs in the transition of brome mosaic virus RNAs from translation to replication was suggested in Galao et al. (2010; Figure 2C).

Plant antiviral defense based on the PB component AGO1 and its slicer activity can be vulnerable to viral silencing suppressors. For example *Arabidopsis* AGO1 has been shown to be a target of the 2b silencing suppressor of the cucumber mosaic virus (genus *Cucumovirus*). Cucumber mosaic virus 2b co-localizes with AGO1 to PBs and directly blocks AGO1's slicer activity both *in vitro* and *in vivo* (Zhang et al., 2006). AGO1 inactivation is mediated also by the turnip crinkle virus capsid P38 which can mimic AGO-binding GW-repeat proteins (Azevedo et al., 2010) and sweet potato mild mottle virus (genus *Ipomovirus*) P1 protein which blocks target RNA binding to preassembled AGO1 (Kenesi et al., 2017). AGO1 degradation has been reported to be mediated by PVX P25 (Chiu et al., 2010), beet western yellows virus (genus *Polerovirus*) P0 (Baumberger et al., 2007; Bortolamiol et al., 2007) and tomato ringspot virus (genus *Nepovirus*) coat protein (Karran and Sanfacon, 2014). In spite of the important role of AGO2 in antiviral RNA silencing *in planta* (reviewed in Carbonell and Carrington, 2015), it is not known whether it associates e.g., with PBs or SGs. Different types of stresses direct human AGO2 to SGs (Detzer et al., 2011) and it is a component of mammalian PBs (Sen and Blau, 2005; Hubstenberger et al., 2017).

Some recent studies link the antiviral defense response both to translational repression and PBs. Recovery is a phenomenon during which host plants recover from viral symptoms. The amount of polysome-associated viral transcripts is reduced and the amount of PBs is increased in plants recovered from tobacco rattle virus (genus *Tobravirus*) infection proposing an important role for PBs in the elimination of vRNAs (Ma et al., 2015). Translational repression is involved also in recovery from tomato ringspot virus infection (Ghoshal and Sanfaçon, 2014). Activation of a plant nucleotide-binding site leucine-rich repeat (NB-LRR) protein by the tobacco mosaic virus p50 replicase protein fragment can induce an antiviral response against PVX and turnip crinkle virus in *N. benthamiana* (Bhattacharjee et al., 2009). During this antiviral response PVX transcripts are translationally repressed and the number of PBs increases significantly in the affected cells (Meteignier et al., 2016). Although the NB-LRR response, UV stress and RNAi against certain transcripts all induce translational repression and PB formation in plants, the mechanisms leading to enhanced PB assembly are distinct. While induction of RNAi increases the number of PBs and the presence of silencing suppressors like P19 inhibits PB induction, PB formation during the NB-LRR response against PVX is not dependent on RNA silencing (Meteignier et al., 2016). Regardless of the mechanism that promotes PB formation, their increased assembly most likely reflects the high concentration of translationally repressed mRNAs.

## siRNA body proteins and virus infection

siRNA bodies are RNA granules associated with the synthesis of tasiRNA and the amplification of the RNA silencing signal. Plant proteins found in siRNA bodies are listed in Table 3. Many plant viruses interfere with siRNA body functions in order to suppress their contribution to siRNA production. Some viruses such as rock bream iridovirus (genus *Megalocytivirus*), a fish DNA virus, (Zenke and Kim, 2008) and sweet potato chlorotic stunt virus (genus *Closterovirus*; Kreuze et al., 2005), encode class I RNase III enzymes which have RNA silencing suppression capacity. Class 1 RNase III endoribonuclease (RNase3) of sweet potato chlorotic stunt virus contains a dsRNA binding domain and a Mg^2+^-dependent catalytic domain for dsRNA cleavage (Kreuze et al., 2005). Its expression in sweet potato as a transgene promotes the accumulation of sweet potato feathery mottle virus (genus *Potyvirus*; Cuellar et al., 2009). Sweet potato chlorotic stunt virus RNase3 co-localizes to siRNA bodies via interaction with SGS3 (Weinheimer et al., 2016). It is likely that the virus uses SGS3 to target its RNase3 to dsRNA synthesized by RDR6 to cleave them into sRNAs which are too small to be used in RNAi. The potyviral protein VPg is, among its other functions, a suppressor of sense-mediated silencing that localizes to siRNA bodies and interacts with SGS3 to benefit virus infection (Rajamäki et al., 2014; Cheng and Wang, 2017). The turnip mosaic virus VPg carries out its silencing suppression function by targeting SGS3 and its binding partner RDR6 directly to degradation via the 20S proteasome (Cheng and Wang, 2017). As a result the production of vsiRNA in the siRNA-bodies will be inhibited. SGS3 functions are disrupted also by other viruses. For example, tomato yellow leaf curl virus (genus *Begomovirus*) V2 protein interacts directly with SGS3. Mutational analysis showed that this interaction is required for V2 silencing suppression function (Glick et al., 2008). Plantago asiatica mosaic virus (genus *Potexvirus*) TGBp1 (P25) co-localizes to siRNA bodies and inhibits dsRNA synthesis by the SGS3/RDR6 complex (Okano et al., 2014). Furthermore, yeast two-hybrid and bimolecular fluorescence assays revealed that the p2 silencing suppressor protein of the rice stripe virus (genus *Tenuivirus*) interacts with SGS3 suggesting that it could act through inactivation of the RDR6/SGS3 pathway (Du et al., 2011).

**Table 3:** Plant siRNA body proteins and their animal counterparts.

**Protein component in plants**	**Animal homolog**	**Function**	**References**
RNA-DEPENDENT RNA POLYMERASE 6 (RDR6)	no known homologs	Amplification of the RNA silencing signal, synthesis of trans-acting RNAs	Kumakura et al., 2009
SUPPRESSOR OF GENE SILENCING 3 (SGS3)	no known homologs	Amplification of the RNA silencing signal, synthesis of trans-acting RNAs	Kumakura et al., 2009
ARGONAUTE 7 (AGO7)	Argonaute family	Processing of trans-acting RNAs	Jouannet et al., 2012
AtALKBH9B	ALKBH5	m6A demethylase, partial association also with PB markers	Martinez-Perez et al., 2017

Some viruses interfere with the RNAi amplification step by inhibiting RDR6 functions. For example, the βC1 protein of tomato yellow leaf curl China betasatellite (family *Geminiviridae*) up-regulates the expression of *N. benthamiana* calmodulin-like protein REGULATOR OF RNA SILENCING (Nbrgs-CaM) which is an endogenous silencing suppressor. In turn, up-regulation of Nbrgs-CaM leads to the suppression of RDR6 gene expression resulting in both PTGS suppression and symptom induction (Li et al., 2014). Downregulation of RDR6 mRNA accumulation is also achieved by the silencing suppressor HCPro of sugarcane mosaic virus (genus *Potyvirus*; Zhang X. et al., 2008). The P6 protein of rice yellow stunt rhabdovirus (genus *Nucleorhabdovirus*) interacts with rice and *Arabidopsis* RDR6 *in vivo* and affects the production of secondary siRNAs by RDR6 (Guo et al., 2013). In conclusion, interference with siRNA body functions to inhibit the RNAi amplification phase seems to be widely used by both RNA and DNA viruses.

## Future prospects

The nucleocytoplasmic compartmentalization of eukaryotic cells paved the way to the development of an extensive post-transcriptional regulatory network at the level of RNA decay. In addition to the core mRNA decay machinery, regulation is provided by several sRNA pathways (siRNAs, miRNAs), NMD and mechanisms targeting RNAs displaying specific secondary structures. An interesting hypothesis of the exaptive origins of regulated mRNA decay in eukaryotes has been set forward by Hamid and Makeyev (2016). They propose that the emergence of RNAi, NMD, and mRNA decay based on the recognition of specific RNA structural elements to aid in non-self RNA recognition coincided with the expansion of RNA viruses. Only later these mechanisms adapted to regulate cellular mRNAs. Out of the currently recognized plant virus genera roughly 70 % represent (+)RNA viruses, 9% (-)RNA and 9% dsRNA viruses (Dolja and Koonin, 2011). RNA viruses are overrepresented in plants as only about 12% of plant virus genera are DNA viruses and retroelements and viroids still add to the number of pathogenic RNA molecules within plant cells. Taking into account the vast number of RNA viruses in plants it is likely that more discoveries linking plant virus infection, antiviral defense, innate immune signaling and cellular RNA biology will be made in the future. This is exemplified by a recent finding of a novel type of RNA-targeting antiviral defense by Drosha-type Class 2 RNase III nuclease (Aguado et al., 2017). Drosha, which has evolved to function in the biogenesis of miRNAs in eukaryotic organisms, and related RNase III enzymes are translocated into the cytoplasm upon RNA virus infection. There they elicit unique RNA-targeting antiviral activity against viruses belonging to families *Togaviridae* and *Flaviviridae* via binding to certain stem-loop structures on vRNA thus hindering the activity of the RNA-dependent RNA polymerases (Aguado et al., 2017). When a pre-miRNA sequence is nested into the flavivirus-like turnip crinkle virus RNA it also becomes processed by an RNase III activity indicating that this enzyme is translocated from the nucleus to the cytoplasm upon virus infection also in higher plants. The authors propose that this particular type of RNase III-mediated antiviral defense conserved in mammals, arthropods, fish and plants is independent of the other known eukaryotic antiviral defense mechanisms and may have preceded them. Furthermore, Martinez-Perez et al. (2017) recently discovered a novel mechanism that regulates plant viral infection via N^6^-methyladenosine (m^6^A) modifications of vRNA. Human immunodeficiency virus (genus *Lentivirus*) and hepatitis C virus (genus *Hepacivirus*) use this mechanism to regulate viral gene expression and infection (Tirumuru et al., 2016) and infectious particle production (Gokhale et al., 2016), respectively. The m^6^A abundance on alfalfa mosaic virus (genus *Alfamovirus*) genomic RNA is regulated by *Arabidopsis* AtALKBH9B, which carries m^6^A demethylase activity (Martinez-Perez et al., 2017). Its silencing restricts the alfalfa mosaic virus infection. *In vivo* AtALKBH9B co-localizes with siRNA body component SGS3 and the NMD component UPF1 and shows a partial association with the PB component DCP1 both in healthy and infected *N. benthamiana* tissues. An NMD-mediated regulation of alfalfa mosaic virus infection was proposed by the authors. Infection by another bromovirus, the cucumber mosaic virus, was not affected by the knockdown of AtALKBH9B. Interestingly, AtALKBH9B interacts with alfalfa mosaic virus coat protein whereas there is no interaction with the cucumber mosaic virus coat protein. Therefore, the authors found it possible that AtALKBH9B exerts its regulatory function on virus infection via coat protein interaction.

While many investigations have paid attention to the amplification of vRNA in replication complexes and post-replication fate of vRNA in relation to gene silencing, the virus-host interactions regulating the replicated vRNA through the rest of the cellular RNA regulatory network is a research area that only recently gained attention in plant biology. Viruses have evolved to regulate host RNA metabolism and SGs, PBs, siRNA bodies and other subcellular structures associated with it in order to maintain the cellular environment optimal for robust viral gene expression and avoid the elimination of vRNA. Apart from the core proteins, the protein composition of RNA granules formed by different plant viruses likely varies. Methods based on the enrichment of the RNA granules via centrifugation and subsequent affinity purification via antibodies or specific affinity tags have been developed for plant (Hafrén et al., 2015), animal and yeast (Wheeler et al., 2017) RNA granules. A recent study in which PBs were purified by fluorescence-activated particle sorting from human epithelial cells followed by characterization of the associated mRNAs revealed that PBs mainly accumulate translationally repressed but not decayed mRNAs (Hubstenberger et al., 2017), suggesting a more pronounced role for PBs in accumulating translationally repressed mRNAs and less so in mRNA decay than previously anticipated. This study shows the importance of biochemical purification of the RNA granules in obtaining more detailed information about their protein and RNA composition as well as functions, giving one possible direction were to go to understand also the pro- and antiviral activities associated with RNA granules of plant origin.

Even with the best studied animal viruses there are still substantial knowledge gaps in understanding the viral interactions with RNA granule components as discussed by Lloyd (2016). For example the molecular mechanisms how enteroviral proteases control the translational apparatus, SGs, PBs and the activation of innate immune response by cleaving their key regulatory components have been worked out extensively but many open questions related to the mechanistic roles of these key components in RNA granule assembly and mRNP recruitment as well as in innate immune activation still remain. While many SG, PB, and siRNA body proteins of plant origin are known and some plant viral interactions with them have been described there is much to discover in this insufficiently studied area. Similarly the ways how plant viruses utilize RNA granule components for their benefit, how plants defend themselves against viruses via RNA granules and how viruses counter the RNA granule-associated antiviral actions are likely versatile. As an example of these complex interactions, a recent study indicated the presence of an autophagy-associated cargo adaptor protein NBR1 in potyvirus-induced RNA granules in turnip mosaic virus infection (Hafrén et al., 2017). Bulk autophagy was found essential for plant fitness and virus accumulation, whereas an antiviral role was identified for selective NBR1-mediated autophagy targeting the RNA silencing suppressor HCPro. NBR1 co-localizes together with HCPro and AGO1 to granule structures which proposes that HCPro is likely targeted to the RNA granule context. Interestingly, a specific form of autophagy called granulophagy is targeting both SGs and PBs in yeast (Buchan et al., 2013). Potyvirus-induced granules, which contain both SG and BP markers (Hafrén et al., 2015), were proposed to be targeted by granulophagy (Hafrén et al., 2017). Potyviral proteins VPg and to a lesser extent 6K2 were found to resist autophagy-related HCPro turnover. The authors propose that these complex interactions between potyvirus-induced granules, potyviral proteins and autophagic degradation form another layer to the co-evolutionary arms race between potyviruses and their host plants. This example together with the others discussed in this review show that beyond RNA silencing and its counter defense there is a plethora of interactions connected to post-replication vRNA regulation in plant cells to be discovered during the coming years.

## Author contributions

KM: Writing the text; AL: Writing the text, drawing the figures; MP: Writing the text, Compiling the tables.

### Conflict of interest statement

The authors declare that the research was conducted in the absence of any commercial or financial relationships that could be construed as a potential conflict of interest.
